# Recreation providers' and end-users' twofold experiences: perception and knowledge about recreation programs in the Ba-Phalaborwa Local Municipality, South Africa

**DOI:** 10.3389/fspor.2026.1713807

**Published:** 2026-10-16

**Authors:** T. B. Ngobeni, M. J. Malema

**Affiliations:** Department of Sports, Recreation & Exercise Science, Faculty of Community and Health Sciences, University of the Western Cape, Cape Town, South Africa

**Keywords:** recreation, recreational activities, recreational experiences, recreational opportunities, youth with disabilities

## Abstract

**Introduction:**

The current study sought to provide insights from two stakeholder groups: recreation service providers and youth with disabilities. People with disabilities continue to face various challenges related to their involvement in recreational programs, particularly in rural areas. Recreational service providers appear to offer programs that can accommodate all individuals; however, the reality is different. This study explored the recreational program experiences of youth with disabilities and recreation program service providers' perceived knowledge.

**Methods:**

This study used a qualitative interpretive research approach with an exploratory design. Participants included local recreation service providers and youth with disabilities in Ba-Phalaborwa, Limpopo Province. Data analysis followed the social model framework and was conducted inductively. Data were collected through semi-structured, open-ended interviews. The study followed ethical standards and adhered to research governance principles and guidelines.

**Findings/Results:**

The findings provide significant insights from two different perspectives. Recreation service providers report having a unique understanding of how recreational programs should be implemented for people with disabilities. Their understanding demonstrates the inclusive practices and steps required for inclusivity. On the other hand, youth with disabilities reported their experiences, highlighting the lack of consultations during activity planning. Their experiences reflect a lack of consideration within their communities and limited access to basic facilities that would enable barrier-free recreational programs.

**Discussion:**

The implications of the present study involve various stakeholders, including local municipalities as a primary liaison for joint efforts, so they can meaningfully influence the livelihoods of people with disabilities and their recreational participation. In conclusion, meaningful participation in recreation requires collaboration between recreation service providers and community members, particularly people with disabilities.

## Introduction

This study is part two of an earlier topic that focused on the lived leisure experiences of young adults in South Africa (1). This study therefore extends the focus by exploring young adults with disabilities and leisure service providers to gain insights into their lived realities and expectations of the services provided. Lack of knowledge about recreational opportunities is a common barrier to participation in recreational activities among people with disabilities (2). The researchers argue that in South Africa, people with disabilities are often marginalised because of insufficient recreational opportunities, including where to access them and how to assert their rights. The researchers observed that recreational activities are misunderstood and often referred to as a luxury. Fancourt et al. report that once people with disabilities know where to look and how to navigate recreational opportunities, their participation is likely to increase (3). This study advocates inclusion as a facilitator of participation in adaptive recreational opportunities.

Inclusion is the process of creating opportunities and environments that meet diverse needs, abilities, and backgrounds, ensuring equal access and participation for all people in leisure and recreation programmes (4). Mohammadi, suggests including people with disabilities in every activity and encouraging them to take on roles like their peers without disabilities (5). According to Masanovic, proper adaptive equipment can make recreational activities accessible to people with disabilities (6). Unfortunately, limited programs exist because adaptive equipment is costly (7). Community members may not fully understand inclusion or its implications for belonging and acceptance; misunderstandings often surround implementing inclusivity (1, 3). These factors can often be challenging to address. Therefore, recreation service providers need the knowledge and resources to offer creative, inclusive, and adaptable recreational programs.

Adaptive recreational programs allow people with physical disabilities to engage in recreational activities similar to those of people without disabilities (1, 8). Wright and Titus indicated that service providers need to make recreational opportunities available to people with physical disabilities (9). The authors noted barriers such as physical, social, and managerial barriers that impact access to recreational opportunities for individuals with physical disabilities (9). The premise of the current study is that recreational activities and accessibility should be designed to meet the specific needs of people living with physical disabilities and accommodate them in ways that support their abilities. Cordts et al. stated that in the disabled community, needs are the same as those of their counterparts; differences include, but are not limited to, modes of transportation and degrees of independence in self-care, which may vary; however, basic needs are shared by all (10).

Mkwelo et al. reported that people with disabilities' leisure and recreational activities are no different from those of able-bodied persons (11). These activities can include watching TV, exercising, socialising, and other leisure-time physical activities (11). Similarly, Malema et al. noted this in a study which focused on the leisure needs as perceived and experienced by deaf adults in the North West province of South Africa (12). Malema et al. argued that despite unequal access to leisure and recreation, one must never assume that people with disabilities do not participate in, or desire to engage in, leisure and recreational activities (12). It was suggested that leisure and recreational service providers should not assume that people with disabilities must be “boxed” because of their inability to perform certain actions; instead, they should work to ensure diverse leisure needs are met (12). Khanyile et al. highlighted a similar notion, reporting a need for recreational hubs to maintain high-quality services that meet users' expectations (13). The lack of structured activities makes high-quality recreational services necessary, as it prevents participants from knowing in advance which programs recreational hubs offer (13). Therefore, providing recreational opportunities is just as important as a person's quality of life.

The current study asserts that recreational opportunities in local communities have expanded over time for all individuals, regardless of age, gender, or disability. The positive outcomes of recreational activities generally come through physical activity, but secondary benefits, such as psychological growth and personal development, should not be overlooked. This is applicable to this study because recreational activities help create and sustain a positive, inclusive social environment in which social and life skills can be fostered and strengthened. Carbone et al. assert that participating in recreational activities can encourage inclusion, minimise conditioning, optimise physical function, and improve overall well-being among people living with physical disabilities (14). Therefore, strategic action at the national, regional, and local levels must emphasise the importance of recreational activities.

Recreational opportunities are a significant component of an individual's life. It can be argued that lack of participation in recreational activities is one of the most significant risk factors for high mortality caused by chronic diseases (14). People with physical disabilities are at risk of having more health-related problems compared to those without disabilities (2). People who are less active have a 20%–30% higher risk of death than more active people. Physical inactivity can lead to fatigue and depression and increase dependence on others. It also increases the risk of heart disease, diabetes, high blood pressure, stroke, pressure sores, weaker bones and several cancers (15). Therefore, recreation officers and relevant stakeholders need to assess and re-evaluate how services and experiences are planned and delivered for people with physical disabilities in their communities (16). Participating in recreational activities can enhance the psychological well-being of people with disabilities by providing opportunities to form friendships, develop a self-identity, and foster meaning and purpose in life (17).

In the context of the present study, we argue that people with disabilities should be encouraged to access mainstream recreational hubs in their communities. Oldridge-Turner et al. indicate that promoting recreation through coherent strategies and policy development is a significant step toward improving community quality of life (18). This gap is prevalent in South Africa, where policy documents often fall short in implementation at the community level. This implementation has a huge impact on people with disabilities, as they are marginalised in mainstream society. Community-based hubs could promote inclusion by removing physical and emotional barriers, addressing stigma, and correcting misconceptions that people with disabilities do not want to participate in recreational activities (16).

## Theoretical framework

This study is underpinned by the social model of disability to respond to how and why people with disabilities are marginalised and the service providers' experiences in offering recreational programs. The social model of disability has influenced public policy on disability issues, seeking to challenge and change beliefs, attitudes, policies and environments that have excluded people with disabilities (19). The researchers recognise the model's limitations; however, the present study aligns with three social domains: environmental, attitudinal, and organisational, which were used deductively during analysis. These barriers act as barriers and facilitators to participation in recreational activities. Identifying these barriers helps facilitate social integration for people with physical disabilities (20). The social model is relevant to the study because of social disparities in South Africa. Despite efforts to address historical inequalities, South Africa still faces challenges such as limited access to sports facilities (21). While the White Paper on Sport and Recreation and the National Sport and Recreation Plan (NSRP), introduced in 2011 and 2012, respectively, acknowledge sport's role in promoting personal and social development, most communities have yet to realise this reality (22, 23).

The current study asserts that the social model of disability challenges attitudinal, physical, systemic, communication and technological barriers created by society. The researchers note that removing these barriers does not automatically lead to participation. In this study, this relates to recreational activities planned and implemented by relevant stakeholders and experienced by people with disabilities. The social model of disability does not deny the impact of an individual's disability; rather, it places the responsibility on society to accommodate people with disabilities instead of expecting the individual to accommodate society (24).

Removing these barriers requires expertise, collaboration, advocacy and legal support. The present study suggests that everyone can play a role in educating themselves about these barriers and learning how to create new pathways to ensure society is accessible and inclusive. The social model of disability not only identifies society as the main cause of disability but also provides a framework for understanding how society behaves toward people with disabilities (25). It also identifies barriers people with disabilities experience and how these barriers can be removed, minimised, or mitigated through other forms of support. This model aims to reduce barriers to inclusion and inform disability professionals (20).

## Research problem

The level of participation in recreational activity among people with disabilities is affected by a complex set of barriers and facilitators unique to this population. This challenge is particularly notable in rural areas, the context of the present study. The current study perceives and observes that people with disabilities are marginalised because of the under-resourced facilities in rural areas of Limpopo province. It is a premise of the present study that people with disabilities in South Africa continue to face barriers that prevent them from participating in recreational activities of their choice (26). These barriers contribute to low participation among young people with physical disabilities. Perceived and reported barriers include attitudinal barriers, limited information about available opportunities, limited knowledge and expertise, inadequate community facilities, limited access to programs, and insufficient accommodation for various disabilities (11, 27). Physical barriers include buildings with no wheelchair access, inadequate lighting that reduces visibility for people with low vision, sidewalks too narrow for walking aids, or doorknobs that are difficult to grasp for people with arthritis (28).

According to Leffel, changes in physical space include structural revamps to accommodate wheelchair users, accessible beach mark mats, and low-sensory quiet hours at the grocery store (28). This study argues that architects, designers, town planners, and the Department of Sport, Arts and Culture in South Africa need to prioritise accessibility so every individual can enjoy recreational opportunities, rather than accommodating only a select few. Therefore, the current study sought to address this gap by exploring and describing the subject matter. This study explored the recreational program experiences of youth with disabilities and service providers' perceptions of program delivery. The present study sought to answer the research question: How do the experiences of recreation service providers and people with physical disabilities differ regarding the programs offered in their communities?

## Methodology

This study adopted an interpretive paradigm focused on understanding the world through participants' experiences. It focuses on how knowledge about participants and cultures is generated (28, 29). This study used a qualitative approach with an exploratory design. An exploratory design seeks to explain participants' lived experiences (30). This helped the researcher gain new insights, discover new ideas, and increase knowledge of the phenomenon (31). The objectives of the study were (a) to explore the lived experiences of youth with disabilities during recreational program planning and participation; (b) to describe the experiences of recreation service providers regarding program planning.

### Participant's profile

The total number of people living with physical disabilities registered at the identified centres for people with disabilities was 125. The study used purposive sampling to recruit and select five recreation service providers and twelve youth with physical disabilities. Only recreational service providers and young people living with physical disabilities formed part of this study. Participants could speak and understand information independently. Table 1 and 2 report the participants' profile who participated in the study. Participants from the Ba-Phalaborwa Local Municipality were also included in this study. According to Mweshi and Sakyi, purposive sampling is a non-probability sampling method used to select participants based on population characteristics and the study's objectives (32).

**Table 1:** Demographic profile of stakeholders.

Pseudonym	Gender	Characteristics
RSP1	Female	Sport promotion officer
RSP2	Female	Director of sport and recreation
RSP3	Male	Sport promotion officer
RSP4	Male	Sport development officer
RSP5	Male	Community sport liaison

**Table 2:** Demographic profile of youth with disabilities.

Pseudonym	Gender	Centers	Age	Disability
YwD 1	Female	A	19	Spinal cord injury due to car accident
YwD 2	Female	A	22	Spinal cord injury due to gunshot
YwD 3	Female	A	18	Congenital disability
YwD 4	Female	B	26	Stroke
YwD 5	Female	B	31	Stroke
YwD 6	Female	A	30	Spinal cord injury due to gunshot
YwD 7	Male	B	24	Stroke
YwD 8	Male	B	19	Spinal cord injury due to gunshot
YwD 9	Male	A	18	Spinal cord injury due to car accident
YwD 10	Male	A	25	Stroke
YwD 11	Male	A	30	Spinal cord injury due to gunshot
YwD 12	Male	B	20	Congenital disability

### Procedure

This study used semi-structured, face-to-face interviews as the primary data collection method. The researchers used an interview schedule to collect data. An invitation letter was sent via email to centers for people living with physical disabilities and the Ba-Phalaborwa Local Municipality to inform them about the purpose of the study. Each recruited participant signed an informed consent form 24–48 h before data collection, and the researcher arranged interviews at times convenient for the participants. Participation in this study was voluntary, and the researcher clearly explained the study's purpose. With participants' consent, the researcher audio-recorded the interviews. Interviews lasted 30–50 min. Interview questions included the following themes: (a) perspective of recreation opportunities; (b) the challenges encountered during recreational activities; and (c) accessibility of recreational facilities for people with physical disabilities.

### Data analysis

The study data were audio-recorded and transcribed verbatim. Thematic analysis was used to analyse the data. According to Braun and Clarke, thematic analysis is a method for analysing, identifying, and reporting patterns within the data, including organising and describing the dataset in detail and interpreting various aspects of the research topic (33, 34). Figure 1 below illustrates the six steps involved in completing the thematic analysis process as highlighted by Braun and Clarke (33, 34). First, the researcher immersed herself in the data, re-reading the transcripts alongside the audio interviews (33, 34). This was followed by the researchers drafting initial codes. The researchers then met thereafter to discuss the themes and codes developed during the analysis. This step ensured no duplication of themes and proper alignment with the study objectives.

**Figure 1:**
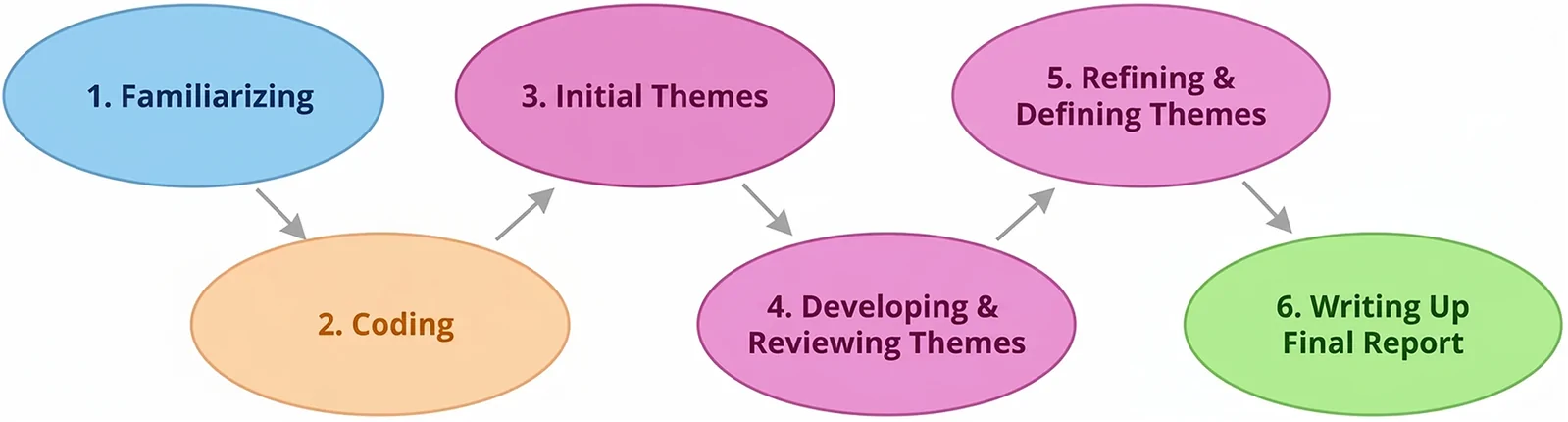
Braun and Clarke (33, 34).

Silverman suggests noting initial themes and concepts, identifying sub-themes, applying a thematic framework, and making meaning by creating thematic charts (35). To analyse the data in this study, the researchers used open coding on transcribed data in ATLAS.ti 9. The researchers identified broad topics and themes from each interview transcript, then cross-coded across all interviews. The first and second authors discussed data analysis and emerging themes and reached consensus on the themes and sub-themes, which contributed to the study's trustworthiness. Both researchers conduct research and community engagement projects on disability, and the first author is a resident of Phalaborwa town. She acknowledged the potential influence her role may have on the research process. Her personal experience of the township and the centres where data was collected could have influenced the researcher's judgement. To address this potential bias, the first author kept a reflexive journal, which the authors later discussed during analysis.

### Rigour of the study

Rigour refers to the degree to which qualitative research reflects participant perspectives and the context under investigation, ensuring the quality of the study (36). According to Gunawan and Bang, trustworthiness provides evidence of rigour for readers to assess the outcomes (37, 38). This study's rigour domain included credibility, transferability, dependability, data triangulation, and member checks. Credibility refers to the confidence in how well data address the intended focus (38, 39). The researcher triangulated by examining participants' experiences from different perspectives. The researcher compared the findings with the study's literature review to support the findings (39). The researcher conducted member-checking with participants to enhance the credibility of the findings. After completing and transcribing the individual interviews, the researcher returned the transcripts to participants, including people with disabilities and recreation providers under Ba-Phalaborwa Municipality, to verify that their views were accurately captured.

This process allowed participants to review the transcribed data and confirm the accuracy of the information they had provided. The researcher established transferability by providing evidence that the study's findings could apply to other contexts, situations, times, and populations (37). The researcher ensured the methods were clear and that the findings were logically linked to the conclusions. Dependability refers to data stability over time and under different conditions (38). To achieve dependability, the researcher reported each process in detail so external researchers could replicate the study and obtain similar findings.

### Ethics

In this study, the researcher sought ethical approval from the University of Western Cape via the Human Social Science Research Ethics Committee (HS21/7/38) and from Ba-Phalaborwa Municipality and centres for people living with disabilities. Participants were informed about the study's purpose. The information sheet, permission letter and informed consent forms were translated into Xitsonga for those who did not understand English. Before the interviews, participants were informed that participation was voluntary and that they could withdraw at any time without prejudice. The researchers maintained participant anonymity by not using real names. The researchers stored transcripts and audio recordings in an encrypted, password-protected file. This study did not intend to harm participants, and no risks were reported during the research process.

## Findings

The present study identified five key themes: (a) Inclusion practice, (b) Service providers’ headaches, (c) Frustrations in recreation engagement, (d) Availability of facilities and (e) Recommendations to bridge the gap. A total of fifteen participants, including (a) five recreation providers and (b) twelve young people, both female and male, were represented in the findings from this study. Verbatim quotations illustrate the findings.

### Theme one: inclusion practice

Inclusive recreational activities require recreation providers to be qualified, knowledgeable, and committed. Before implementing any program, providers should plan and organise it regularly to meet participants' needs. When designing program environments for people with disabilities, these spaces should benefit everyone. Below are comments highlighted by key informants: “*For participants who are using wheelchairs, we adjust tables according to their height, and they have light rackets*” RSP 2. Furthermore, RSP 4 said: “*Recreational activity provided for people living with disabilities is Para volley; it is like a volley, but it is different because it is meant for people who are physically challenged, who have got one limb, wheelchair users*”. The sentiment expressed shows that service providers and end-users experience inclusive recreation practices differently. According to the White Paper on Sport and Recreation of South Africa, adaptations within the physical and social environment are essential to facilitate participation and promote inclusion among people with physical disabilities (23). Unfortunately, these statements do not guarantee participation; however, the findings show a thought process that must be unpacked properly to ensure successful implementation. These findings offer a contrasting account between recreation service providers' perceptions and the experiences of people with disabilities. This demonstrates a gap and disconnect between end-users and service providers.

The experiences of young people with disabilities reveal an interesting perspective about the recreational services offered to them. An unfortunate incident reported by YwD stated that: “*Most of them, the community initiated them [recreational programs] for themselves where they buy their own equipment*”. This was further explained by YwD10 reporting that “*They always overlooked the need to involve persons with disability in sport as we are deemed unfit to participate due to our physical ailments*” YwD 10. The findings above reiterate the attitudinal barrier domain from the social model of disability. The findings above reiterate the social model of disability's position that the barriers emerge from the community rather than the individual (20). These expressions align with the attitudinal domain; the recreation service providers report a positive attitude by recognising the need to adapt resources to meet participants' activity needs. Without support from all stakeholders, achieving inclusive recreation remains challenging. Everyone is entitled to equal access to those benefits, and considering the needs of people with disabilities when designing all policies, programs, services, facilities, and environments will make participation in sports and active recreation more inclusive and appealing for everyone.

### Theme two: service provider's headache

Service providers are often confronted with challenges in offering recreational programs for people with disabilities, particularly due to limited funds, equipment and adaptive resources. Participants reported: “*I am not happy about the recreational participation of people living with disabilities because the number keeps on decreasing every day; the majority of people living with physical disabilities are not willing to participate in sports and recreation, and some do not have information about the benefits they will acquire through recreational activities, hence others are not interested…*” RSP 2. Additionally, RSP 3 expanded on this notion, reporting that: “*We once had a challenge whereby some of the participants could not access the facility because there were no ramps for wheelchair users. The participants had to stay outside, which demotivated other participants to do their level best*” RSP 3.

These sentiments show unequal opportunities for end users. The notion is further reported by RSP 4, who mentioned that: “*Four players play goal ball at a time, two on the other goal post and two on another one goal post. Therefore, we make use of a ball which is a 5-size ball, but it has a ring inside, so the way it is being played is a matter of rolling it on the surface that is going to the other goal post, so when it gets inside the post, we count it as a goal*”. The findings show that recreation providers face challenges in delivering recreational activities. Recreation service providers need to collaborate with the caregivers, guardians, and parents of people with disabilities. The results in this theme highlight that those organisational barriers, as a domain within the social model of disability, must be conquered (20). Recreation service providers need to ensure facilities are accessible to everyone by embracing universal design principles.

### Theme three: frustrations in recreation engagement

Lack of recreation programs for people with disabilities is a major concern that needs to be addressed. Additional challenges include perceptual barriers, limited media coverage and inadequate equipment. Participants shared their lived experiences, stating that: “*The challenge we face is that we are marginalised from these activities because of our disability, and as such, we feel left out; we only see people without disabilities participating*” YwD 11. These sentiments are strengthened by YwD 8, who report that: “*When local municipalities provide recreational programs available for the community, they do not include people living with physical disabilities during their planning phases*”.

These challenges can have a significant negative impact on the potential success of prospective persons with disabilities. The participants shared the following experience: “*The community where we live is not conducive to sport, as there are no sporting grounds. The other challenge is that we do not have funding, which is allocated to enable equipment, as far as equipment is concerned, because, as people living with physical disabilities, we cannot use the same type of adapted equipment that is slightly different from that used by people without disabilities due to our abilities. Therefore, we are unable to participate in recreational activities like other people living without disabilities*” YwD 6. The findings above highlight the frustrations and injustices faced by people with physical disabilities in their communities. These findings align with the environmental domain barrier in the social model of disability (20). People with disabilities' sustained motivation to participate in recreational activities is influenced by their ability to remove the barriers they encounter. Environmental factors, including physical and social environments, partly determine people with disabilities' participation in recreational activities.

### Theme four: availability of facilities

Participation in recreational activities cannot be possible without adaptive equipment and accessible programs. Buildings were originally designed with the needs of nondisabled people in mind, making them inaccessible and less user-friendly for individuals with physical disabilities. The participants highlighted that: “*In accordance with departmental norms, we do not have our own facilities, so we outsource the facility from local municipalities. For example, if we are to host a Para volley tournament, we will look for a hall which will also cater for wheelchair users. We make sure that everything is in order and that wheelchair ramps are accessible so that the participants can easily access the hall freely*” RSP 5. Adding to this notion, RSP 2 said: “*Some of the facilities do have ramps, but some do not. Usually, when we host an event for people living with physical disabilities, we try to look for a facility which will be accessible and have ramps*” RSP 2.

The findings above demonstrate that recreational service providers' inability to offer impactful and engaging activities is affected by the facilities where the programs are hosted. Limited facility access directly affects user-friendliness. In response to the experiences of people with disabilities, they report their lived experiences as follows: “*I would not recommend it for anyone with physical disabilities because there were plenty of staircases, and the tables and chairs were not adjustable or lowered, so I had to get myself seated at the table. This became a problem because I loved playing chess; I would always find my way through. Another thing is that people are not always willing to help when they see you struggling. If you can’t find your way through, you would often find yourself disheartened and discouraged from pursuing your passion*” YwD 3.

Participants indicated that ramps in their area help people with physical disabilities access recreational facilities more easily. However, this remains a concern, as service providers must outsource these facilities, making them available only during tournaments. The findings align with the organisational barrier domain within the social model of disabilities (20). Lack of accessibility is a major reason why some people living with disabilities have lower levels of engagement in recreational programs. Moreover, access to youth recreation programs and opportunities is important for communities to promote recreational activity and enhance social belonging.

### Theme five: recommendations to bridge the gap

Provision of access to opportunities for people with disabilities must be addressed equally alongside opportunities for their non-disabled peers. These recommendations are context-specific and may not be universally applicable; therefore, exercise caution when implementing them. RSP 3 shared their perception, indicating that: “*What we must keep in mind as recreation officers and community members is that people with physical disabilities should be treated like everyone; they must not feel sidelined because of their condition. There should be a development of more suitable programs for people with disabilities, and we need to encourage people with disabilities to participate in sports*”. Educating people about recreational activities can enhance participation among people living with physical disabilities. The participants shared the following comments: RSP 4 said: “*It is very important to educate the caregivers about the availability of recreational programs for people with disabilities. This education needs to start from the household level, where these participants stay up until they go to the play space or the court, because they need to be well integrated in the community and in the household to show that they are accepted*”.

Recreation service providers expressed genuine concern about ensuring that people with physical disabilities participate in recreational programs in their communities. The findings offer a tangible solution to ensure that people with disabilities are not marginalised. Meanwhile, youth with disabilities shared their recommendations based on their lived experiences and encounters. They provided their responses; YwD 1 suggested that: “*The department of sports and local municipalities must allocate funds to our centres to help with recreation programs. Most people have potential and are willing to participate in sports, but there is no equipment or facilities. People need to be taught, and they need to have knowledge about people living with disabilities that would be my recommendation*”*.*

There is a genuine need for recreation providers to assess and re-evaluate how programs for people with physical disabilities are designed and delivered in rural communities. YwD 12 mentions that: “*I can say that there is a need to design or develop appropriate opportunities for people with physical disabilities, which includes access to recreational facilities, because our facilities are not accommodating to us wheelchair users*”*.* The findings revealed that awareness campaigns should be conducted regularly to motivate people with disabilities to participate in sports and recreational activities. The findings above indicate that, to improve accessibility and identify suitable recreational activities, people with disabilities must know about relevant facilities and available opportunities. This information should be made widely accessible. Therefore, promoting information about barrier-free recreational facilities and activities is important to create opportunities for participation without difficulty.

## Discussion

This study aimed to explore and describe recreational opportunities for young people living with physical disabilities in Phalaborwa, Limpopo province, South Africa. The study reported five key themes: Inclusion Practice, Service Providers’ Challenges, Frustrations in Recreation Engagement, Availability of Facilities, and Recommendations to Bridge the Gap. The participants were represented in their own voices as recreational service providers and youth with disabilities, both male and female. Key findings revealed that recreational opportunities for people with physical disabilities are a challenge among people with physical disabilities. These challenges include limited adaptive programs, accessibility issues, recreational barriers and limited recreational programs. The participants indicated that discrimination and exclusion contribute to their lower levels of participation compared to people without disabilities.

Building on this, inclusive recreational activities acknowledge diverse skills and abilities, recognising that sports can be modified to accommodate players. For example, tee-ball evolved from baseball to accommodate different levels of understanding and ability. Lack of inclusive activities or the provision of one-on-one training is a structural constraint (1). Muthivhi, argues that inaccessibility of facilities is the main reason why people living with disabilities do not participate in recreational activities in rural communities (11, 40). The findings consequently suggest that accessibility cannot be understood solely as the provision of individual accommodations. Rather, equitable participation requires systematically incorporating accessibility into the planning, design, and management of transport, recreation, and tourism environments. This perspective highlights the distinction between formal equality and substantive accessibility.

The participants indicated that there is still a gap in opportunities for participation among people living with disabilities. The results show a continued need to provide recreational opportunities that accommodate everyone. According to the Americans with Disabilities Act, people with disabilities have the right to participate in recreational activities and to receive benefits comparable to those of people without disabilities (41). Copestake et al. argue that people living with disabilities reported that the places and spaces they wish to visit are often inaccessible, with barriers such as the lack of clean and adequate disabled toilets and changing facilities (42).

Furthermore, the findings of this study revealed that young people with physical disabilities are not fully involved in recreational opportunities due to societal barriers, challenges during participation, limited recreational programs and inaccessible facilities. These challenges hinder them from engaging in recreational activities of their choice, and as a result, they feel discriminated against. This shows that needs analysis should be considered, as it forms the basis for developing a strategic plan for recreation participation. Participants also indicated that they often have difficulty finding desired recreational activities because of a lack of suitably trained coaches and volunteers. This finding is similar to Arral, which highlights that people with disabilities face societal barriers and that no amount of positivity will remove them without educating people about inclusive programs and accessibility (10, 11, 43). Therefore, to become an all-inclusive and accessible society, we need to change the way we perceive people living with disabilities and collaborate with them in our community. By doing this, we can break down these barriers.

In addition, the findings revealed that limited recreational programs are a major concern that needs to be addressed, as they are demotivating and make participants feel excluded from society. Participants also indicated that they experience different barriers and facilitators to recreational participation. These findings support the study conducted by Ginis et al. which indicates that society perceives people with physical disabilities as incapable of participating in recreational activities due to their condition (44). This perception alone has a significant negative impact on the potential success of prospective disabled athletes. Kim et al. also supports the view that most people living with physical disabilities get discouraged from participating in recreational activities due to limited recreational facilities, and accessible recreational facilities motivate participation (45).

Moreover, the findings revealed that accessibility is still a challenge for people with physical disabilities because some of the facilities do not have ramps for wheelchair users. As a result, wheelchair users find it hard to attend tournaments and events. Dickson et al. support this finding by outlining that accessibility and inclusivity for people with physical disabilities are worldwide issues, and that only those without physical disabilities fully enjoy themselves (1, 46). Edwards et al. and Ginis et al. also indicate that society views a person with a disability as someone incapable of doing things independently (44, 47). Arral, states that people living with disabilities encounter societal barriers, and no amount of positivity can eradicate these barriers without educating people about inclusive programs and accessibility (43). Therefore, to become an all-inclusive and accessible society, we need to change how we perceive people living with disabilities and collaborate with them in our community; by doing so, we will break down these barriers.

According to the social model of disability, everyone can play a role in educating themselves about these barriers and learning how to create a positive environment so society is as accessible and accommodating as possible. The findings of this study outline that young people living with physical disabilities experience different barriers and facilitators for recreational participation, which could increase their risk of developing secondary health conditions. Diaz et al. claim that knowledge about barriers and facilitators of recreational participation is needed because people are not aware of what it entails to provide recreational activities for people with physical disabilities (1, 4, 48).

Finally, the findings revealed that recreational participation can change and empower people with disabilities to realise their full potential and advocate for societal changes to eliminate attitudinal barriers. Through recreational activities, people with physical disabilities acquire vital social skills and develop independence (48). The results show that the availability of recreational opportunities plays a significant role in encouraging physical activity and engagement in various recreational activities of choice. Barber indicates that being active not only keeps people fit and healthy but also provides emotional, intellectual and social benefits (49). Regular recreational activity reduces antisocial behaviour, encourages healthy growth and development, and enhances self-concept. Adaptive recreation programs and equipment offer people with disabilities many benefits, including exercise to improve health, increased self-esteem, and opportunities to experience new and exciting activities (49). Moreover, the participation of people with physical disabilities alongside other community members can help reduce societal barriers (12). The findings reflect a critique of liberalism, especially the assumption that providing similar recreation and tourism facilities to everyone automatically ensures access for all.

### Recommendations and limitations for the study

Local municipalities should establish a team that includes both people living with physical disabilities and those without disabilities from each community. Including both groups ensure inclusivity and avoids creating the impression that people with disabilities are being isolated. The local government should raise awareness about the rights and dignity of people with disabilities and combat stereotypes, prejudices and lack of information about the available recreation programs. Training recreation staff, people with physical disabilities, and caregivers should be a central goal for recreation providers to promote inclusive participation. This can lead to better socialisation and a better understanding of how to make recreational activities accessible and inclusive. Local municipalities should improve and maintain facilities such as stadiums and community halls to ensure accessibility and promote inclusion. Communities should have a sports unit for people with disabilities where they can access information about upcoming tournaments and request equipment and training. The present study is based on a small sample, which limits the generalisability of the findings. However, this limitation does not diminish the key findings reported by the current study.

## Data Availability

The raw data supporting the conclusions of this article will be made available by the authors, without undue reservation.
